# Supplementary material for: Fragment-based screening identifies inhibitors of ATPase activity and of hexamer formation of Cagα from the Helicobacter pylori type IV secretion system
Source: Sci Rep. 2019 Apr 24;9:6474. doi: 10.1038/s41598-019-42876-6 (PMC6482174; doi:10.1038/s41598-019-42876-6)

Supplementary tables, figures and legends

Title : **Fragment-based screening identifies inhibitors of ATPase activity and of hexamer formation of Cagα from the *Helicobacter pylori* type IV secretion system**

Authors : Tarun Arya, Flore Oudouhou, Bastien Casu, Benoit Bessette, Jurgen Sygusch and Christian Baron*

Affiliation : Department of Biochemistry and Molecular Medicine, Faculty of Medicine, Université de Montréal, Québec, Canada

***Corresponding Author:** E-mail: christian.baron@umontreal.ca

Figure S1

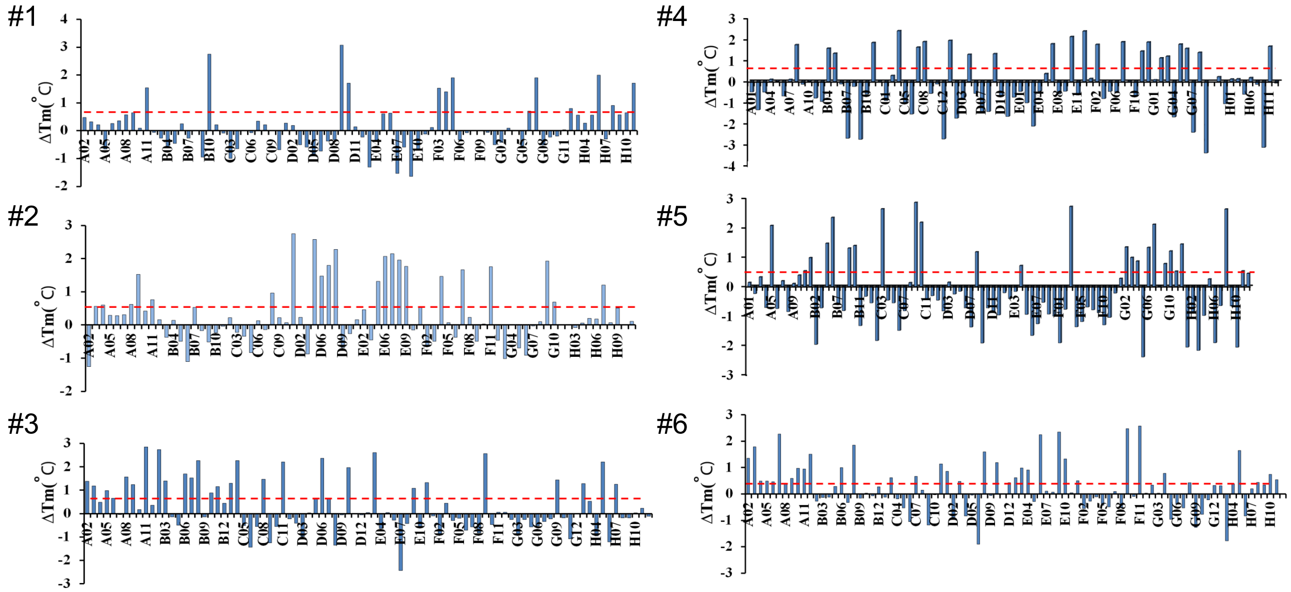

Supplementary Figure 1. Differential scanning fluorimetry screening of fragment libraries to identify Cagα binding molecules. Melting temperatures of Cagα in the presence of a library of 505 fragments dispensed from six master plates (#1 to #6). Differences of T_m between Cagα alone and in the presence of fragments are shown as (ΔT_m). Molecules are considered as stabilizing if they change the T_m value higher than twice the standard deviation of reactions with Cagα alone (red line). To focus on the molecules that have the strongest effects we chose a stricter cutoff and only followed up on the 16 molecules that increased the T_m value by more than 1°C (supplementary Figure 2).

Figure S2

	Structure
**A8**	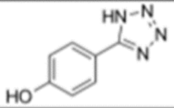
**B5**	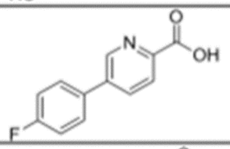
**E7**	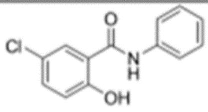
**E10**	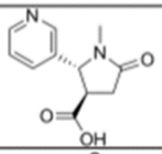
**F12**	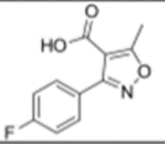
**G2**	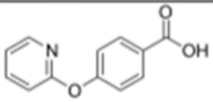
**G6**	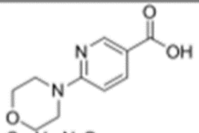 $C_{10}H_{12}N_2O_3$
**G8**	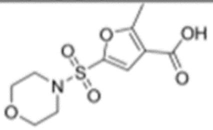

	Structure
**A5**	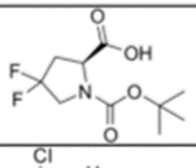
**C3**	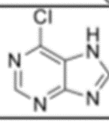
**C9**	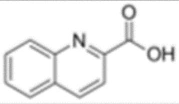
**E4**	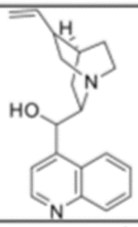
**G6**	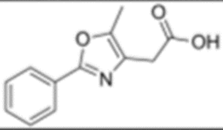
**G7**	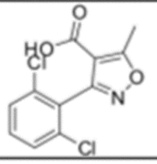
**G11**	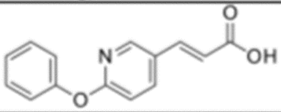
**G12**	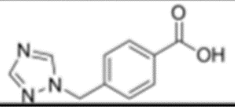

Supplementary Figure 2. Structures of 16 Cagα-stabilizing molecules identified by differential scanning fluorimetry.

Figure S3

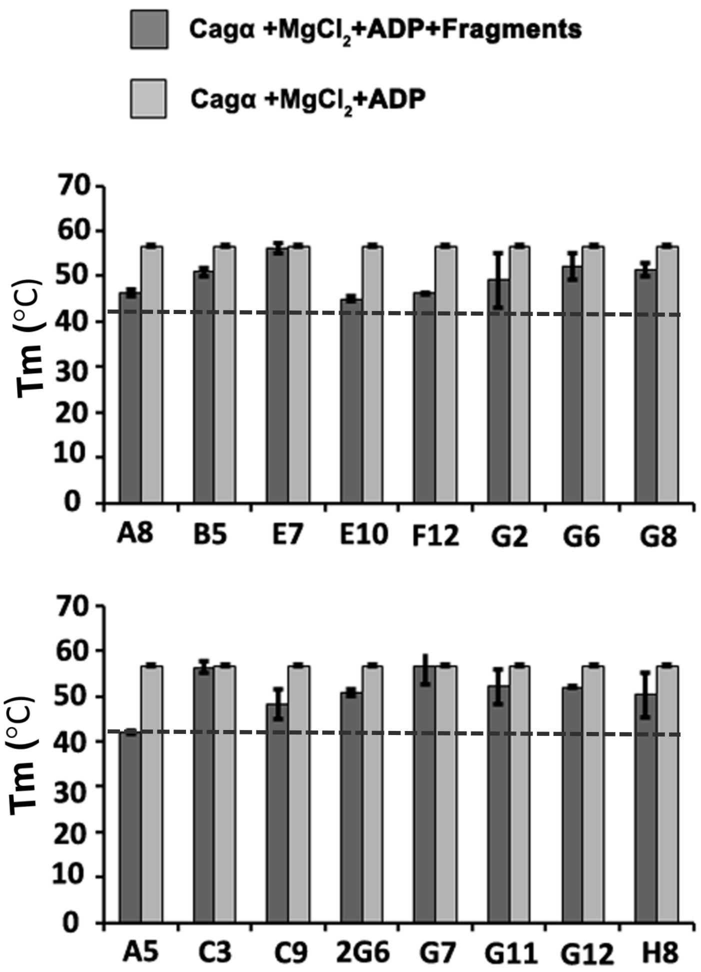

Supplementary Figure 3. Changes of melting temperatures in the presence ADP and Mg^{2+} after co-incubation with 16 Cagα-stabilizing molecules. Melting temperatures of Cagα were determined in the presence of ADP and Mg^{2+} (light grey) with ADP, Mg^{2+} and stabilizing fragments (dark grey) and the melting temperature of Cagα in the presence of Mg^{2+} alone is shown as comparison (dotted line). The data represent the results from three experiments.

Figure S4

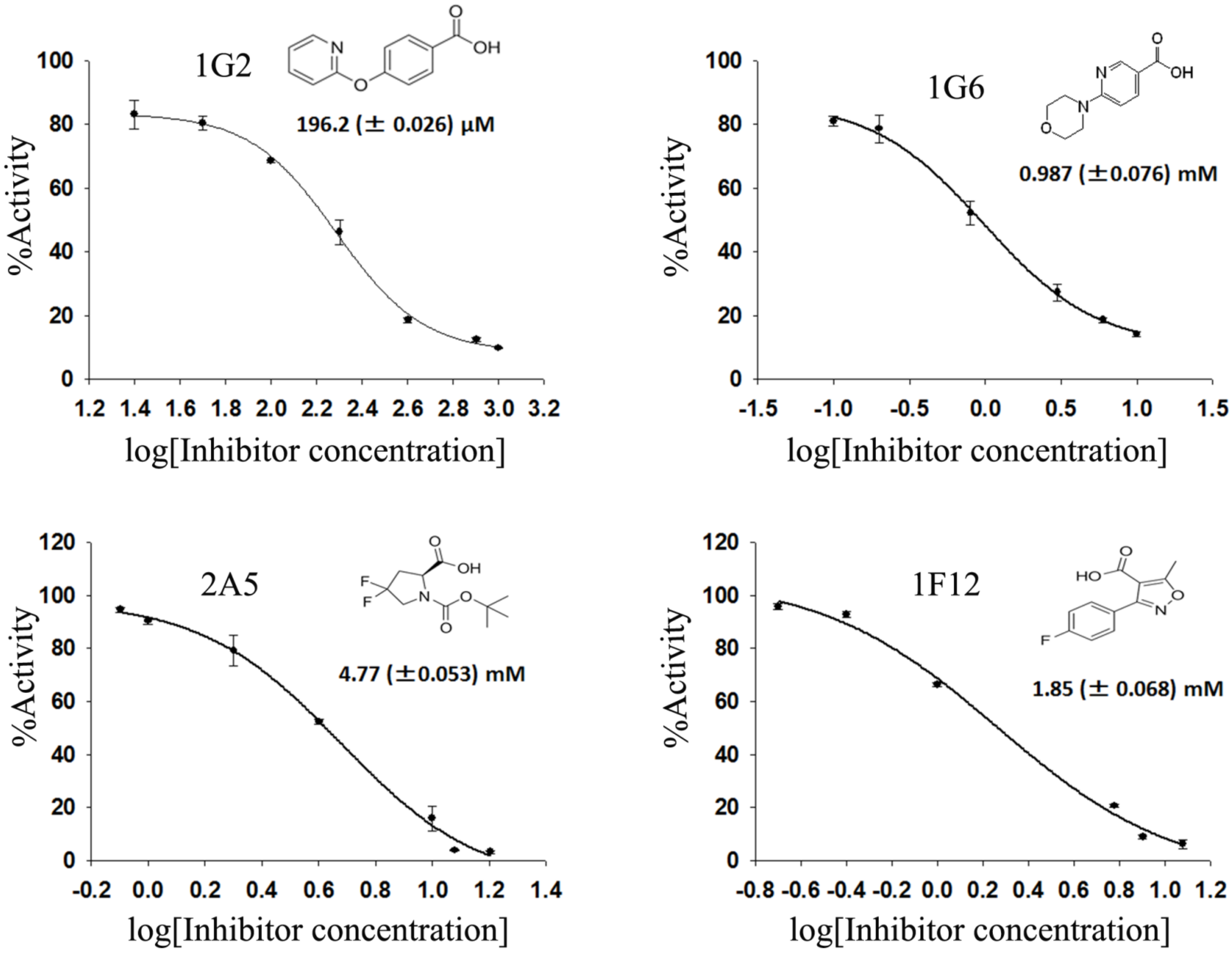

Supplementary Figure 4. Dose response curves of ATPase activity showing IC_{50} values in the presence of four molecules that inhibit the enzyme activity.

Figure S5

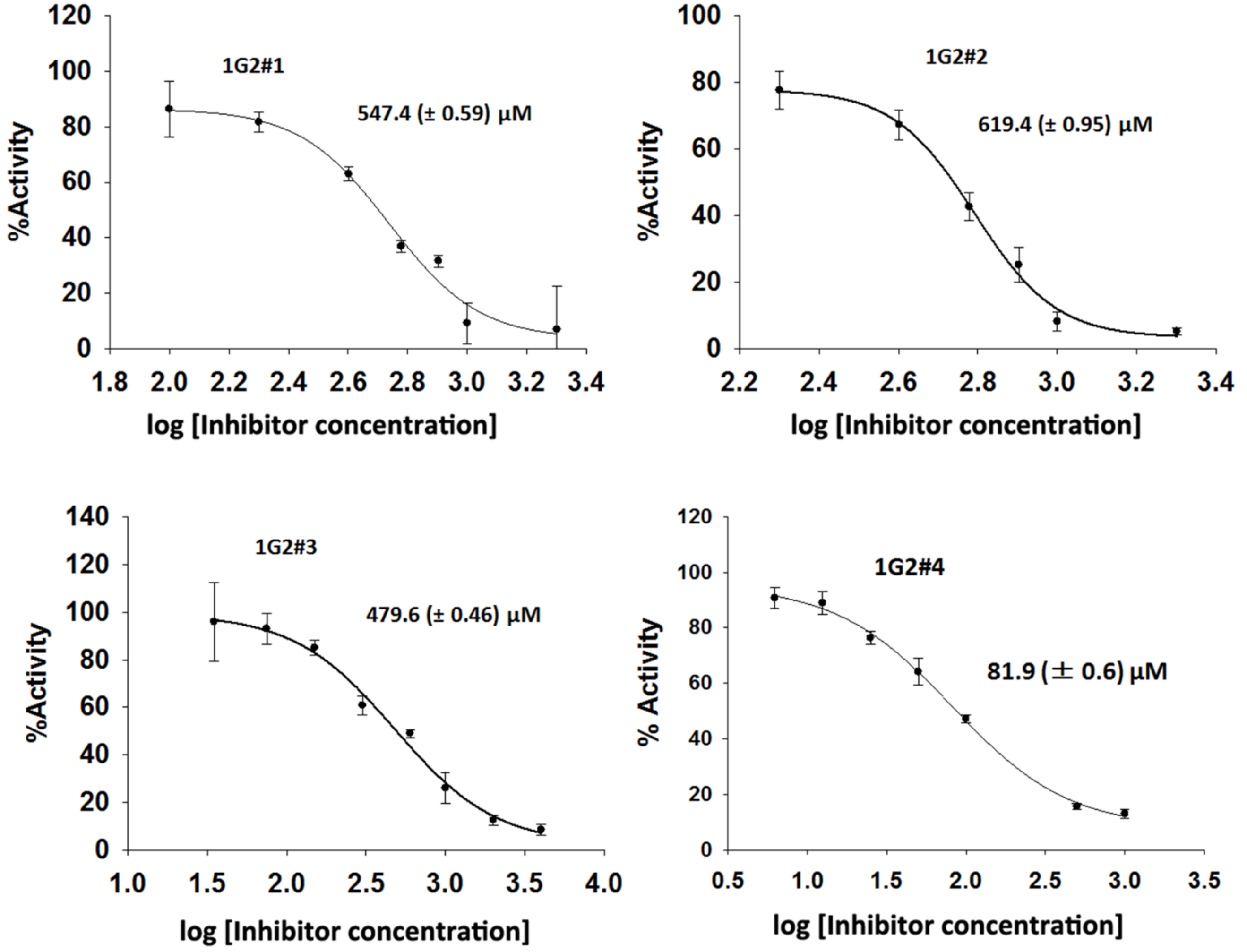

Supplementary Figure 5. Dose response curves of ATPase activity showing IC_{50} values in the presence of four derivatives of molecule 1G2 that inhibit the enzyme activity.

Figure S6

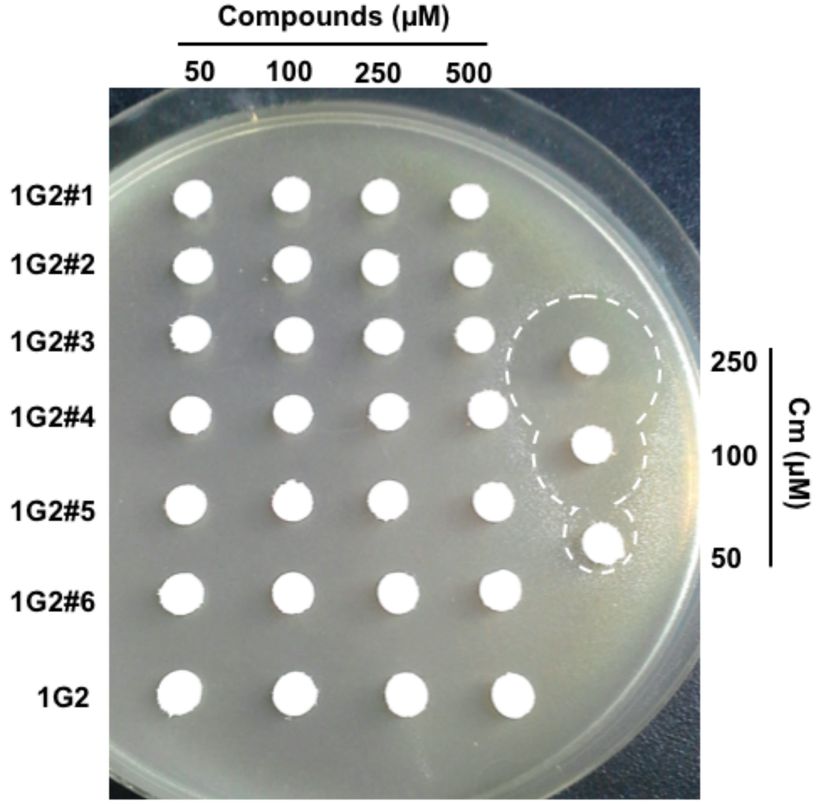

Supplementary Figure 6. Effects of molecule 1G2 and its derivates on *H. pylori* growth. Molecule 1G2 and six derivates (1G2-1 to 1G2-6) were spotted on Whatman paper disks at increasing concentrations (from 50 to 500 µM) and placed on an agar plate inoculated with *H. pylori* 26695. Several concentrations of chloramphenicol (Cm) were tested as control. Growth was observed after 72 h of incubation, the halo around the Whatman paper disks with Cm indicate the inhibition of growth and the inhibition zone is labeled with lines for clarity.

Figure S7

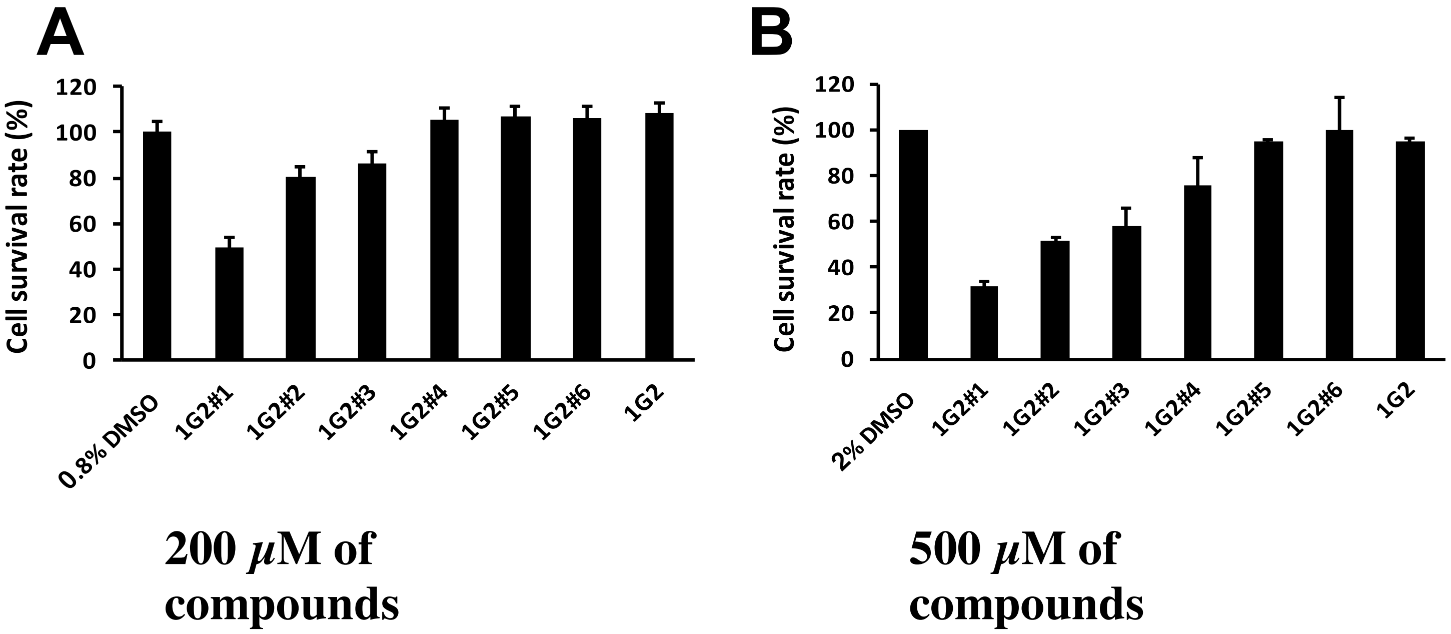

Supplementary Figure 7. **Effects of molecule 1G2 and its derivatives on the viability of AGS cells.** a) AGS cells were incubated with 0.8% DMSO or 200 µM of 1G2 and its derivatives for 24h. b) AGS cells were incubated with 2% DMSO or 500 µM of 1G2 and its derivatives for 24h. Cell survival was measured using the Cell Proliferation Reagent WST-1 kit and cell survival in the presence of DMSO was calculated as 100 %.

Figure S8

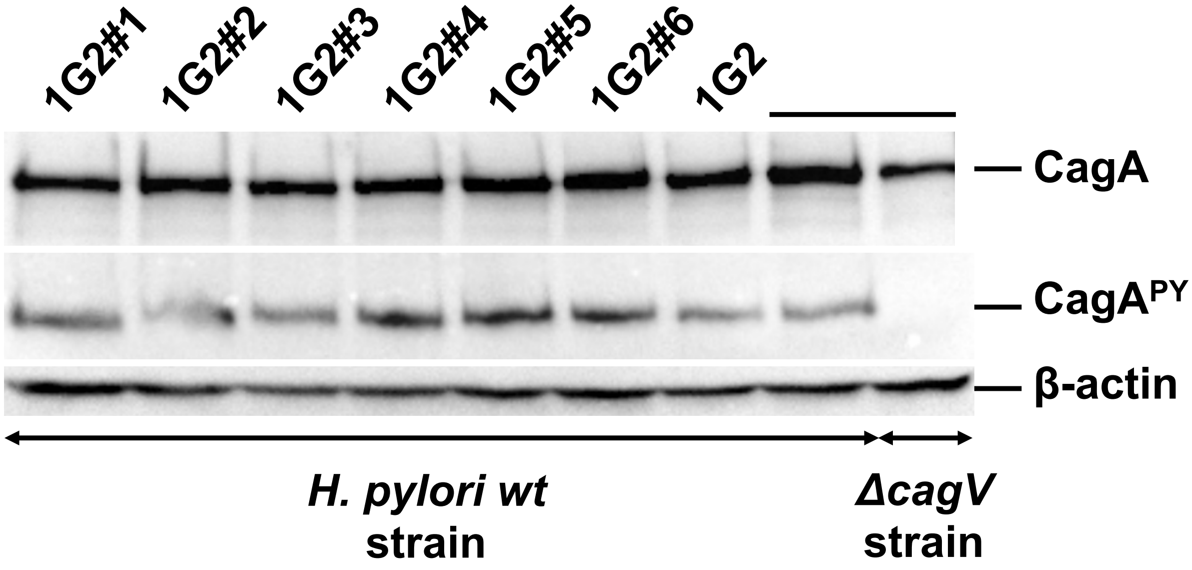

Supplementary Figure 8. **CagA phosphorylation in the presence of molecule 1G2 and its derivates**. *H. pylori* strain *26692* wild type was pre-incubated with 1G2 and its derivatives, followed by infection of AGS cells (lanes 1-7); *H. pylori* wild type without added compound (lane 8) and *H. pylori* strain *26692 ΔcagV* strain (lane 9) were used as controls. Western blotting was performed with anti-CagA (CagA), and anti-phosphotyrosine antibodies ($CagA^{PY}$), anti-β-actin antibody was used as loading control. Western blot analyses were reproduced four times and intensities of the $CagA^{PY}$ signals were quantified (Image Lab™ Software) and normalized using anti-β-actin signals. Statistical analyses (Anova test) do not show significant differences between experimental conditions, except for *ΔcagV* negative control (not shown).

Figure 3 - Original Blots

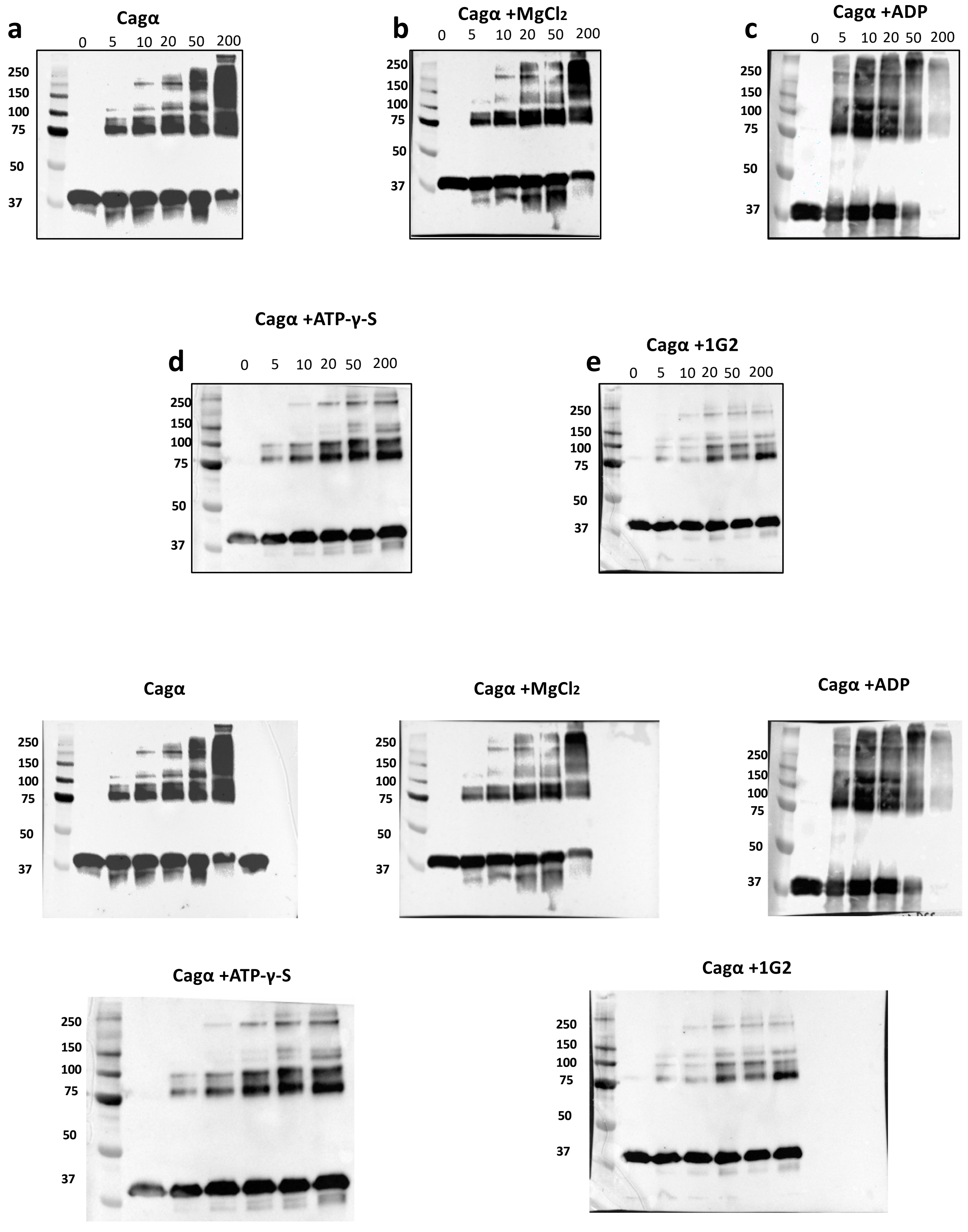

Supplement: Supplementary file 1 — Supplementary figures [file 41598_2019_42876_MOESM1_ESM.pdf]
